# Insanity from the Use of Chloroform during Parturition

**Published:** 1850-08

**Authors:** 


*Insanity from the Uuse of Chloroform during Parturition.*—Dr. Webster related to the Westminster Medical Society (Dec. 15th, 1849) the following case, communicated to him by a professional friend, in consequence of perusing in the *Lancet* a report of the three similar instances he had mentioned at a previous meeting of the society. Only one drachm of chloroform, sprinkled upon a handkerchief, was used; but the effect it produced was so sudden and violent, that the patient, after inhaling, remaining quite insensible, which greatly alarmed the attendants. With the insensibility there was likewise deadly paleness of the countenance; however, she slowly rallied, but had a painful and protracted labor. During several days subsequently, the lady continued in a very nervous condition, although not then actually incoherent, but she soon became so furiously maniacal as to require coercion by a strait-waistcoat. After being insane during many months, the patient gradually recovered her reason, and ultimately got convalescent. Considering it was only from accumulated facts and extensive experience that sound practical knowledge respecting the employment of chloroform in midwifery could be acquired, Dr. Webster then said he had related the present, as likewise the previous examples of insanity following its use, in order to contribute data towards that important object; and he availed himself of the present opportunity to state that he should esteem it a favor if other practitioners would communicate to him any well-marked cases of the same kind, with particulars, which they may have met with during their practice, as he (Dr. Webster) was very desirous of collecting additional evidence upon this interesting subject; of course, on the express understanding that neither the patient's name should be divulged, nor the correspondent in any way compromised, all such communications being considered strictly confidential in regard to individuals.—*Lond. Med. Gaz*, Jan., 1850.

